# Supercritical fluids behave as complex networks

**DOI:** 10.1038/s41467-023-37645-z

**Published:** 2023-04-10

**Authors:** Filip Simeski, Matthias Ihme

**Affiliations:** 1 Department of Mechanical Engineering, Stanford University, Stanford, CA 94305 USA; 2 Department of Photon Science, SLAC National Accelerator Laboratory, Menlo Park, CA 94025 USA

**Keywords:** Thermodynamics, Phase transitions and critical phenomena, Complex networks

## Abstract

Supercritical fluids play a key role in environmental, geological, and celestial processes, and are of great importance to many scientific and engineering applications. They exhibit strong variations in thermodynamic response functions, which has been hypothesized to stem from the microstructural behavior. However, a direct connection between thermodynamic conditions and the microstructural behavior, as described by molecular clusters, remains an outstanding issue. By utilizing a first-principles-based criterion and self-similarity analysis, we identify energetically localized molecular clusters whose size distribution and connectivity exhibit self-similarity in the extended supercritical phase space. We show that the structural response of these clusters follows a complex network behavior whose dynamics arises from the energetics of isotropic molecular interactions. Furthermore, we demonstrate that a hidden variable network model can accurately describe the structural and dynamical response of supercritical fluids. These results highlight the need for constitutive models and provide a basis to relate the fluid microstructure to thermodynamic response functions.

## Abstract (web summary)

Supercritical fluids have local density inhomogeneities caused by molecular clusters. Authors show that the molecular interactions of supercritical fluids, associated with localized clusters, obey complex network dynamics that can be represented by a hidden-variable network model.

## Introduction

Supercritical fluids occur in a wide range of environmental and technological processes, including atmospheric separation on planets in our solar system^1^, biochemical habitats in submarine hydrothermal vents^2^, fluid-phase transfer in subduction zones^3^, and long-term carbon storage in deep sedimentary formations^4^. In addition, chemical processing^5^, energy conversion^6^, and hydrocarbon production^7^ utilize supercritical fluids in daily operations by taking advantage of the strong variations in thermodiffusive properties, such as heat capacity, surface tension, diffusivity, and solubility^8^. Moreover, the microscopic structure of supercritical fluids exhibits local density inhomogeneities^9^, which characterize a transition between two structurally different states: a high-density liquid-like state and a low-density gas-like state^10–12^. These local density inhomogeneities arise from molecular clusters that are embedded among unbound molecules; each cluster is a grouping of fluid molecules that increases the local density of the fluid. To compartmentalize the supercritical state space, different transition boundaries have been proposed, including Nishikawa’s ‘ridge’^13,14^, the Frenkel line^15,16^, the Widom line^11,17^, and the percolation line^18^. Nishikawa’s ‘ridge’ identifies the region of maximum correlation lengths and density fluctuations along the extension of the coexistence curve^13,14^. The Frenkel line relates to the dynamic crossover between a rigid liquid and a non-rigid liquid, associated with the vibrational motion and the ballistic-collisional motion^15^. Recent studies have examined the structural, thermodynamic, and dynamic properties in relation to this crossover condition and confirmed its presence in supercritical water^19,20^. The Widom line separates liquid-like and gas-like states and indicates the condition where the correlation length attains its maximum. It is often associated with the locus of maxima in thermodynamic response functions^21^. Simeoni et al.^11^ measured sound dispersion in high-pressure argon to show changes in structural behavior as one crosses from the liquid-like to the gas-like state. For supercritical water, Sun et al.^22^ investigated the longitudinal current correlation spectra and found that crossing the Widom line leads to a shift from high to low frequencies, indicating a change from liquid-like to gas-like behavior. Neutron-imaging measurements of density fluctuations in supercritical water confirmed the transition from liquid-like and gas-like conditions across the Widom line^23,24^. In the current work, the Widom line is modeled as the line of maxima in the isobaric heat capacity.

In contrast to the Widom line, the percolation line is directly based on the microscopic structure and marks the critical density at which molecular clusters coalesce and transform from micro- to macroscopic dimensions^18^. Percolation theory was applied to identify supercritical liquid-gas transitions in argon^25^, carbon dioxide^26^, and water^27^. For supercritical water specifically, the percolation crossover was verified by neutron diffraction measurements^10^. In one of the earliest applications of percolation theory to analyze the microscopic structure of water, Stanley^28^ put forward a correlated-site percolation model to describe ambient and supercooled water as a percolating network of hydrogen bonds (H-bonds). Subsequently, Campi et al.^18^ applied percolation theory to supercritical Lennard-Jones fluids and identified a percolation line starting at the critical point. Similarly, through network analysis of Monte Carlo simulation data, dos Santos et al.^29^ observed highly clustered H-bond network in supercritical water and, at ambient conditions, a single giant cluster that percolates the system. More recently, lattice-type models have been proposed to explain the relation between the microscopic structure and thermodynamic response functions of supercritical water^30^ and along the critical isotherm of argon^31^. The lattice structure and constant spacing in these models, however, limit their applicability in high-density states.

In this study, we examine the static and dynamic cluster behavior of water across the structural transition line in the supercritical phase. Different definitions of a cluster have been proposed^32^. Perhaps the simplest definition of a cluster is that of a Stillinger cluster^33^, which assigns all molecules within a prescribed radius to the same cluster. This purely geometric definition has gained traction due to its simplicity, but it has also attracted criticism because of its dependence on tunable parameters^34^. Clusters in supercritical water have also been defined based on the properties of H-bonds^35,36^. More rigorously, Hill proposed a cluster definition based on the atomic energy balance between potential and kinetic energy^37^. By extending Hill’s energetic criterion, we seek to define local clusters based on interactions between molecules. The evolving structure of these clusters controls the fraction of fluid in the liquid-like state. By characterizing cluster agglomeration and fragmentation at the crossover, we discover that the microscopic topology and dynamics can be described by a complex network model. The utility of this model is evaluated by comparing predictions of structural properties from molecular dynamics (MD) simulations and experiments, including the cluster size distribution and the connectivity. Finally, we employ this complex network model to analyze the crossover dynamics.

## Results

**Fig. 1:**
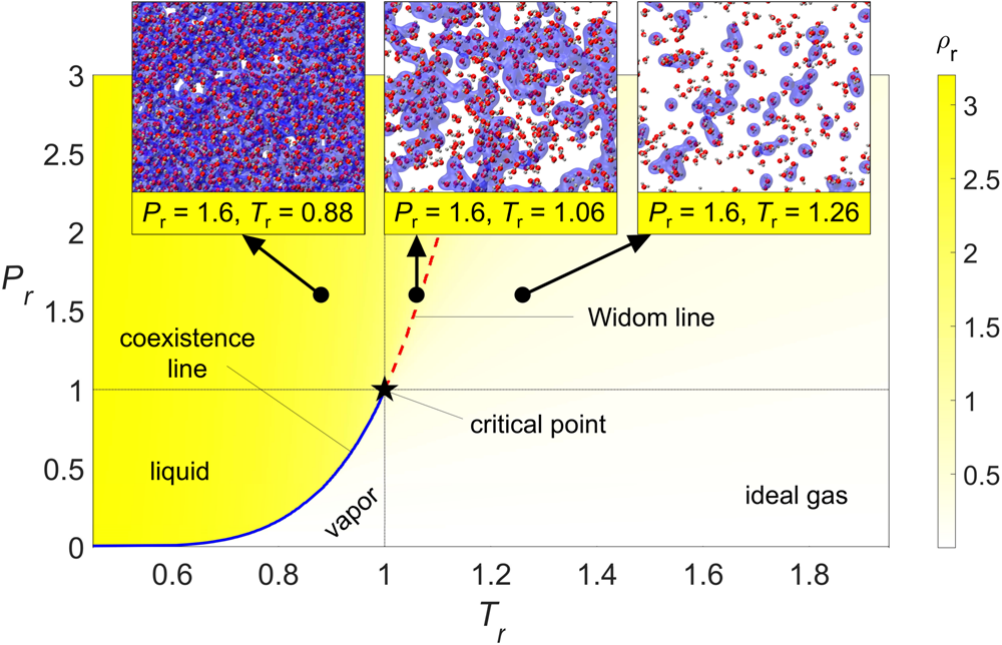
Phase regime diagram for water. The Widom line is shown as a red dashed curve and the critical point is labeled with a black star. Insets show that clusters fragment as temperature is increased: from a liquid-like state (*T*_*r*_ = 0.88), via the Widom line (*T*_*r*_ = 1.06) to a gas-like state (*T*_*r*_ = 1.26). These snapshots show the microscopic structure and clustering in MD simulations of supercritical water at *P*_*r*_ = 1.6; O atoms are represented in red and H atoms are represented in gray. The clusters, defined using Hill’s criterion, are shaded in pale blue.

The properties of supercritical fluids change substantially when crossing the Widom line. This change is illustrated in Fig. 1, showing the reduced density of water, *ρ*_*r*_ = *ρ*/*ρ*_*c*_, as a function of reduced pressure, *P*_*r*_ = *P*/*P*_*c*_, and reduced temperature, *T*_*r*_ = *T*/*T*_*c*_. The critical point is located at *T*_*c*_ = 647 K, *P*_*c*_ = 220.7 bar, and *ρ*_*c*_ = 322.5 kg/m^3^. In supercritical water at temperatures below *T*_*c*_ (Fig. 1, *T*_*r*_ = 0.88), most molecules in the fluid belong to a single cluster, which due to its high density conforms to a liquid-like state. As the temperature is increased, this single cluster fragments into many clusters of all sizes along the Widom line (Fig. 1, *T*_*r*_ = 1.06). Finally, at temperatures exceeding *T*_*c*_ (Fig. 1, *T*_*r*_ = 1.26), only few clusters exist and most molecules belong to a gas-like state that is characterized by disconnected molecules with high kinetic energy. In this phase diagram, the Widom line is determined as the locus of the peaks in isobaric heat capacity from NIST data^38^.

To investigate changes in the microscopic structure of the fluid during the Widom line crossover, we perform MD simulations using the ReaxFF potential^39^. The ability of this force field to predict the structure of supercritical water has been assessed in previous studies^40,41^, and further validation against experimental data is provided in Supplementary Figures 1–4. MD simulations with 8544 molecules were performed in the isothermal-isobaric ensemble (NPT) to study state points spanning reduced pressures from *P*_*r*_ = 1.1–2.8 and reduced temperatures from *T*_*r*_ = 0.46–1.9. A Nosé-Hoover chain thermostat^42^ was used to control the temperature. The chain comprised three thermostats with a damping constant of 10 fs. Similarly, the pressure was controlled by a Nosé-Hoover chain of three barostats^42^ with a pressure damping parameter of 100 fs.

### Cluster identification

The MD trajectories are analyzed to investigate the microscopic cluster structure, which is described via the cluster size distribution, *n*_*c*_(*s*), representing the number of clusters of a given size, *s*, in the system^43^. To avoid ambiguity in the cluster definition, we use Hill’s energy criterion^37^ because it is purely physics-based, numerically robust (see Supplementary Note 1 and Supplementary Figs. 5 and 6), and applicable to general and non-polar fluids. Previously, this criterion was employed to study clustering in supercritical Lennard-Jones fluids^18^. The molecules that belong to a cluster are interconnected with bound pairs. Two molecules, *i* and *j*, form a bound pair if the interaction potential energy exceeds their relative kinetic energy^37^:

1 $${E}_{{{\mbox{pot}}},ij}+{E}_{{{\mbox{kin}}},ij} \, < \, 0.$$

In this work, Hill’s criterion is applied to pair-wise molecular interactions. The interaction potential energy, *E*_pot,*ij*_, between molecular pairs consists of two components: a van-der-Waals interaction and a Coulombic interaction. The ReaxFF force field treatment of interatomic bonds accounts for all vibrational and rotational modes that may affect the structural and dynamical response at supercritical conditions. Moreover, because the van der Waals and Coulombic energetic contributions depend only on the intermolecular distance and not on the molecular orientation, the interactions that lead to bound pairs are isotropic in nature. It is important to note that the current application of Hill’s energy criterion does not include the hydrogen-bonding interaction energy. This assumption is valid because the cluster lifetime is longer than that of individual H-bonds, which was found to be less than 100 fs in the supercritical water phase^44^. Finally, the relative kinetic energy, *E*_kin,*ij*_, is defined based on the velocities of the molecular centers of mass. Based on these energetic interactions, a molecule is assigned to cluster *C*_*m*_:

2 $${C}_{m}:=\left\{i\,|\,(\exists \,j\in {C}_{m})\left[{E}_{{{\mbox{pot}}},ij}+{E}_{{{\mbox{kin}}},ij} < 0\right]\right\},$$

where the clusters are denoted as *m* = 1, 2, …, *N*_*c*_, with *N*_*c*_ being the total number of clusters. A detailed description of this algorithm is presented in Supplementary Note 1. When Hill’s criterion is applied to each pair of molecules in the MD data, the clusters define the microscopic structure of supercritical water (see insets in Fig. 1).

### Self-similar representation of thermodynamic state space

To collapse the fluid properties and microscopic structure for isobaric conditions, we seek a self-similar variable. To achieve this collapse, the data is analyzed in terms of the scaled reduced pressure^45^:

3 $${P}_{r}^{*}={P}_{r}^{{A}_{0}/{A}_{s}},$$

where *A*_*s*_ is the species-specific nondimensional slope of the Widom line at the critical point (*A*_*s*_ = 6.479 for water) and *A*_0_ = 5.51934 is a reference value that is based on the slope of a fluid with zero acentric factor^45^. Further, we introduce a reduced temperature increment with respect to the Widom line, ${{\Delta }}{T}_{r}={T}_{r}-{T}_{r}^{*}$, where ${T}_{r}^{*}=\ln \left({{P}_{r}^{*}}^{1/{A}_{0}}\right)+1$ is the reduced temperature at the Widom line^45^. Combining the scaled reduced pressure and the reduced temperature increment, we introduce the Widom self-similarity:

4 $${{{{{{{\mathcal{W}}}}}}}}=\frac{{{\Delta }}{T}_{r}}{{P}_{r}^{*}}=\frac{1}{{P}_{r}^{*}}\left[{T}_{r}-\ln \left({{P}_{r}^{*}}^{1/{A}_{0}}\right)-1\right],$$

which allows us to collapse fluid properties in the supercritical phase. Specifically, the scaled reduced pressure removes the widening in the peak of thermodynamic response functions with increasing pressure and the reduced temperature increment shifts these functions to collapse. The use of the Widom line for this collapse instead of the percolation line, which would be more appropriate in normalizing of cluster-based properties^18^, is motivated by ease of direct experimental verification of the Widom line and its relation to thermodynamic quantities.

### Complex network model

To connect the cluster distribution to the macroscopic behavior, we hypothesize that the microscopic structure of supercritical fluids can be described as a complex network. While the connection of networks to the molecular organization of fluids has been recognized for a long time^28–31^, previous comparisons have mainly employed lattice-based models, which limited the number of thermodynamic states that could be analyzed in such endeavors. Networks are structures consisting of nodes that are connected through links^46^. In this description, each molecule represents a node, and molecules are connected with a link if they form a bound pair based on Hill’s energy criterion (Equation (1)). Molecules with high kinetic energy are less likely to be bound to other molecules in the system due to their short residence time in each other’s vicinity. The role of molecular energies in the cluster formation implies that links depend on nodal properties. Moreover, mass conservation requires that the total number of nodes remains constant.

To represent the cluster distribution, we consider a complex network model that is based on an exponential random graph^46,47^. Exponential random graphs have been employed to describe a variety of real-world applications, including trade and economic relationships^47,48^, social networks^49^, and conformations of dynamic systems^46^. By considering a complex network model, we represent the probability for the supercritical fluid system to obtain a certain topology, *G*, by^46^:

5 $$p(G)=\frac{1}{Z}\exp \{-H(G)\},$$

where *H*(*G*) is the Hamiltonian of the given topology and $Z={\sum }_{G}\exp \{-H(G)\}$ is the partition function for the system. Starting from Equation (5), and based on the maximum likelihood principle, Garlaschelli and Loffredo^47^ derived a hidden-variable network model where the probability that two nodes are linked is given by:

6 $$f({x}_{i},{x}_{j})=\frac{z{x}_{i}{x}_{j}}{1+z{x}_{i}{x}_{j}},$$

where *x*_*i*_ is the fitness of node *i* and *z* is a variable that controls the network density. The fitness represents the suitability of a molecule to form more links. This network model fulfills the physical requirements: (i) it redraws links in a network at each iteration based on molecular properties and (ii) the total number of molecules in the network is constant.

In this network model, the link density *z* introduces the thermodynamic dependency that is defined via the temperature and pressure conditions of a supercritical fluid state. Because we aim to describe the molecular topology of the supercritical fluid via the network model, we need to express the values of *z* in terms of the thermodynamic conditions of the molecular system. To this end, we first present a relation between the link density *z* and network-topological quantities that can be calculated from the MD data, such as the total number of bound pairs (i.e., links) in the supercritical fluid system. The total number of links, *M*, for the network model is expressed as^50^:

7 $$M=\frac{N}{2}\mathop{\sum }\limits_{k=0}^{\infty }k{n}_{d}(k)=\frac{1}{2}N\bar{k},$$

where *k* is the degree of a node, *n*_*d*_(*k*) represents the distribution of nodes with degree *k*, and *N* is the total number of nodes in the network (i.e., total number of molecules). Finally, $\bar{k}$ represents the average degree of a node^51^:

8 $$\bar{k}={k}_{\max }\int\nolimits_{0}^{1}\int\nolimits_{0}^{1}g({x}_{i})g({x}_{j})f({x}_{i},{x}_{j})\,{{{{{{{\rm{d}}}}}}}}{x}_{i}{{{{{{{\rm{d}}}}}}}}{x}_{j},$$

where ${k}_{\max }$ is the maximum degree of a node in the system. In the thermodynamic limit, ${k}_{\max }\to N$. The integral represents the average probability for a link between any two nodes. Because this integral typically obtains a small value, $\bar{k}$ is always finite. Here, we sample the fitness from a probability density function, *g*(*x*), that is given by a beta distribution:

9 $$g(x;\alpha,\beta )={x}^{\alpha -1}{(1-x)}^{\beta -1}\frac{{{\Gamma }}(\alpha+\beta )}{{{\Gamma }}(\alpha ){{\Gamma }}(\beta )},$$

with $\alpha=\left(\bar{x}(1-\bar{x})-\overline{{x}^{{\prime} 2}}\right)\bar{x}/\overline{{x}^{{\prime} 2}}$ and $\beta=\left(\bar{x}(1-\bar{x})-\overline{{x}^{{\prime} 2}}\right)\left(1-\bar{x}\right)/\overline{{x}^{{\prime} 2}}.$ Here, $\bar{x}=0.6$ and $\overline{{x}^{{\prime} 2}}=0.0686$ are the mean and the variance of the fitness distribution, respectively. These quantities were chosen based on a regression of MD data and were kept constant for all thermodynamic conditions. The relation of the distribution parameters to physical properties requires further investigations.

With equations (7) and (8) yielding a closed analytical solution for the total number of links in the network model, we proceed to connect this model to the MD system. The total number of links, *M*, is assumed as a known quantity and we numerically solve for the corresponding link density *z* that serves as input in generating an appropriate complex network. For the network to converge to a statistically steady state, we adopt the approach of Lin et al.^52^: the presented data is averaged over 15*N* iterations with the self-consistent histogram method^53^. The steps for generating the network are summarized as follows.

1. Assign a fitness value, *x*_*i*_, to each node *i* using Equation (9): *x*_*i*_ ~ *g*(*x*; *α*, *β*).
2. Calculate the probability, *f*(*x*_*i*_, *x*_*j*_), for a link between nodes *i* and *j* using Equation (6).
3. Create a symmetric adjacency matrix by comparing the probability, *f*(*x*_*i*_, *x*_*j*_), against a random number selected from a uniform distribution between (0, 1). No self-links are allowed.
4. From the adjacency matrix, calculate topological properties of the network model.
5. Repeat steps 1-4 for 15*N* times.

Because we aim for the network model to depend only on experimentally measurable quantities, we seek to relate the link density, *z*, to the Widom self-similarity, ${{{{{{{\mathcal{W}}}}}}}}$. Because it has been shown that, in the thermodynamic limit, the hidden-variable model maintains an invariant cluster size distribution in terms of *zN*^54^, we propose the following expression:

10 $$z=\frac{1}{2N}\left(\frac{3}{20}+\exp \left\{{{{{{{{{\mathcal{W}}}}}}}}}^{2}-\frac{13}{2}{{{{{{{\mathcal{W}}}}}}}}+\frac{3}{2}\right\}\right),$$

which is a regression (see Supplementary Fig. 7) of the calculated *z* values from the total number of links, *M*, in the MD data via Equations (7) and (8). Because the link density *z* in Equation (10) is directly calculated from experimentally measurable thermodynamic quantities through Equation (4), this relation allows for a self-contained network model to predict the microscopic structure of supercritical water. We note that the generalization of this representation to other fluids requires further investigation.

**Fig. 2:**
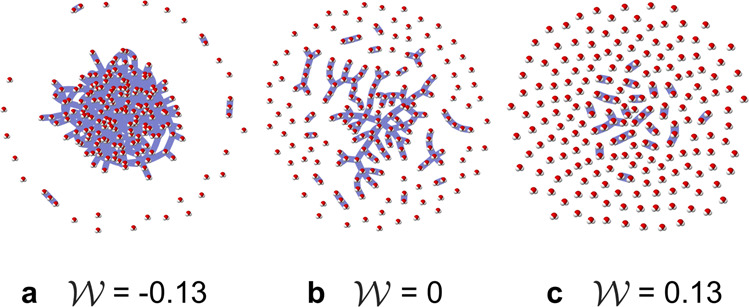
Topology of network model. Snapshots of the hidden-variable model topology at **a**${{{{{{{\mathcal{W}}}}}}}}=-0.13$, **b** 0, and **c** 0.13 in a network of *N* = 200. As the value of ${{{{{{{\mathcal{W}}}}}}}}$ increases, the network exhibits the system-spanning cluster of the liquid-like state, the scale-free cluster size distribution along the Widom line, and the low-density mode of the vapor-like state, respectively. The conditions visualized here correspond (through Equation (10)) to the thermodynamic state points shown in Fig. 1. For visualization purposes, each node is represented with an image of a water molecule. Clusters are depicted by pale blue shading.

Clusters in the network model represent groups of nodes that are connected through links. As shown in Fig. 2, the topology of this network model reproduces the three types of structural behavior observed in the three regimes of supercritical fluids: liquid-like state, percolation, and gas-like state. In what follows, we quantify this structural response. Moreover, we show the capability of this hidden-variable network model to describe the complex physical structure of molecular interactions in the supercritical phase by demonstrating agreement in terms of cluster size distributions, connectivity, and network dynamics.

### Structural properties and topology

**Fig. 3:**
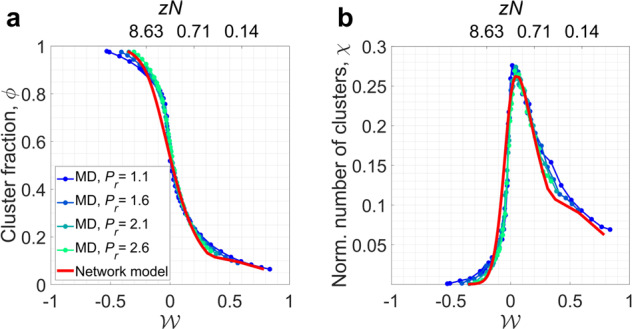
Comparison of structural properties. **a** Cluster fraction and **b** normalized number of clusters in the MD simulations and in the complex network model. The quantities are plotted as a function of the Widom self-similarity ${{{{{{{\mathcal{W}}}}}}}}$ (and corresponding link density, computed from Equation (10)), which collapses these quantities onto a master curve. The similarity in both quantities indicates that the network model predicts the behavior of the real system. Source data are provided as a Source Data file.

To statistically discuss the microscopic structure of supercritical water, we define two quantities of interest: cluster fraction and number of clusters. The cluster fraction, *ϕ*, represents the fraction of fluid that is contained in all molecular clusters (*s* > 1):

11 $$\phi=\frac{{N}_{a}}{N}=1-\frac{{n}_{c}(s=1)}{\mathop{\sum }\nolimits_{s=1}^{\infty }s{n}_{c}(s)},$$

where *N*_*a*_ is the number of molecules that belong to any cluster. Four representative examples of how the cluster fraction evolves as a function of ${{{{{{{\mathcal{W}}}}}}}}$ are shown in Fig. 3a. The collapse of these curves as a function of ${{{{{{{\mathcal{W}}}}}}}}$ implies that a general model may describe the underlying intermolecular interactions across the supercritical region.

The total number of clusters relates how the cluster fraction is distributed in the system by showing whether the liquid-like state is concentrated in a few large clusters or whether there is an abundance of small liquid-like assemblies interspersed with unbounded molecules. The maximum number of clusters occurs when all molecules belong to a different bound pair, i.e., all clusters are the size of two molecules. Therefore, in this work, the normalized number of clusters, *χ*, is defined as:

12 $$\chi=\frac{2{N}_{c}}{N}=\frac{2\mathop{\sum }\nolimits_{s=2}^{\infty }{n}_{c}(s)}{\mathop{\sum }\nolimits_{s=1}^{\infty }s{n}_{c}(s)}.$$

Physical insight is obtained from the normalized number of clusters as a function of ${{{{{{{\mathcal{W}}}}}}}}$ (Fig. 3b), which peaks along the Widom line. This finding is consistent with previous observations of how the number density for clusters in supercritical fluids varies with pressure^55^. At temperatures below the Widom line, there is one large cluster that contains the majority of the molecules in the system, therefore limiting the total number of clusters that can be formed. Once we cross the Widom line, the majority of molecules are unbounded, i.e., they do not belong to a cluster. Because molecules with high kinetic energy are less likely to stay together and form a cluster, there are fewer clusters at temperatures higher than those along the Widom line. This behavior is consistent across the supercritical state space studied here.

We proceed by comparing predictions of the network model with MD data for supercritical water. The cluster fraction (Fig. 3a) predicted from the network model shows good agreement with the cluster fraction variation from MD simulations. As a result, the network model describes the fraction of molecules in the supercritical phase that belongs to a cluster. In a similar manner, the number of clusters observed in the physical system is also reproduced well by the network model. As shown in Fig. 3b, the normalized number of clusters in the network model peaks at ${{{{{{{\mathcal{W}}}}}}}}=0$ and decreases with a similar slope as the MD results for supercritical water. This agreement confirms that the complex network model predicts the structural properties of supercritical fluids at the system level.

**Fig. 4:**
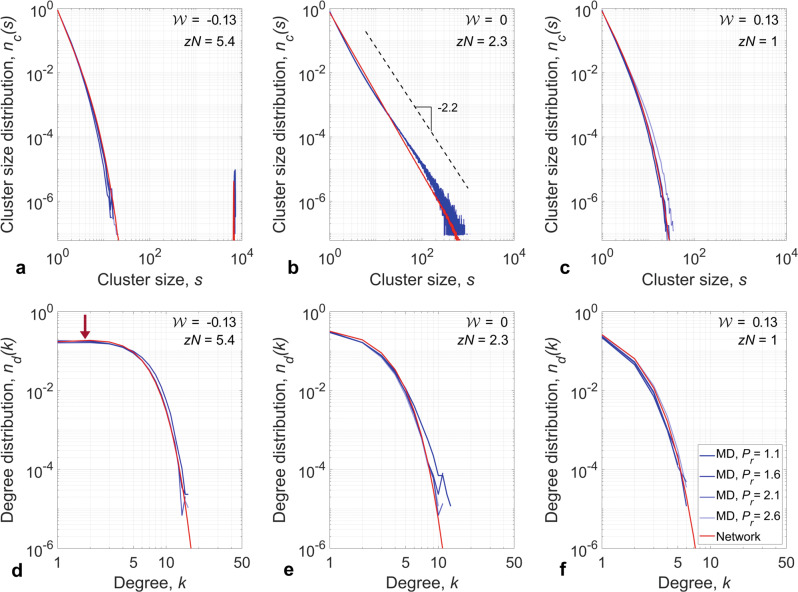
Topology of supercritical fluids. **(top row)** Cluster size distributions and **(bottom row)** degree distribution for supercritical water and the complex network model. Different shades of blue represent isobars, (dark to light) *P*_*r*_: 1.1, 1.6, 2.1, 2.6, respectively. Both quantities exhibit three distinct regimes: (**a**, **d**) liquid-like state (${{{{{{{\mathcal{W}}}}}}}}=-0.13$), (**b**, **e**) Widom line (${{{{{{{\mathcal{W}}}}}}}}=0$), and (**c**, **f**) gas-like state (${{{{{{{\mathcal{W}}}}}}}}=0.13$). Network model agrees well with MD simulations across wide range of thermodynamic conditions. Arrow in **d** marks a peak in the degree distribution. Source data are provided as a Source Data file.

While the cluster fraction and the number of clusters provide statistical information about the microstructure of supercritical fluids, to understand the underlying physics of how these fluids are organized, we study the local density inhomogeneities. Figure 4 shows two descriptors of this density variation: the cluster size distribution, *n*_*c*_(*s*), and the degree distribution, *n*_*d*_(*k*). First, we show that, at each thermodynamic state point, there is an inherent distribution of cluster sizes. The size of the largest cluster depends on the thermodynamic state of the system. This dependence occurs because the molecular bound pairs (i.e., links in the network of intermolecular interactions), defined by Equation (1), break as molecular energies change with thermodynamic conditions. At liquid-like conditions (${{{{{{{\mathcal{W}}}}}}}}=-\!0.13$, Fig. 4a), the cluster size distribution exhibits faster than power-law decay with one very large cluster that is of the same size as the system. At the Widom line (${{{{{{{\mathcal{W}}}}}}}}=0$, Fig. 4b), there are clusters of all sizes and the cluster size distribution obeys a power law. The observation of a power law *n*_*c*_(*s*) ~ *s*^*τ*^ with *τ* = − 2.2 in the cluster size distribution, which was reported by Campi et al.^18^ along the percolation line, confirms that the Widom line closely follows the percolation line for the studied range of conditions. Across this transition, for gas-like conditions (${{{{{{{\mathcal{W}}}}}}}}=0.13$, Fig. 4c), the cluster size distribution shows exponential decay and does not contain clusters that span the system. We extend these results by discovering self-similarity in the cluster size distribution along isopleths of equal ${{{{{{{\mathcal{W}}}}}}}}$. The cluster size distributions remain invariant along each of these isopleths (four different thermodynamic state points are shown for each regime in Fig. 4), an invariance that extends to reduced pressures of at least 2.8.

**Fig. 5:**
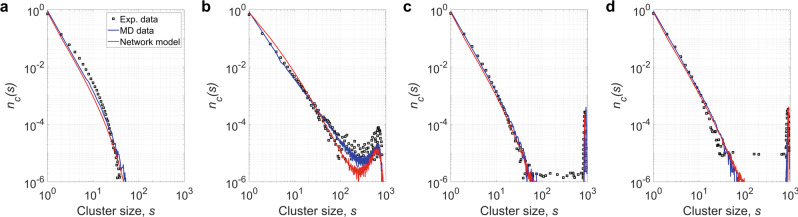
Comparison with experiments. Comparison of cluster size distributions from MD simulations and network model to experimental data of Bernabei et al.^10^ for four conditions: **a** *P*_*r*_ = 1.13 and *T*_*r*_ = 1.04, **b** *P*_*r*_ = 2.26 and *T*_*r*_ = 1.04, **c** *P*_*r*_ = 6.79 and *T*_*r*_ = 1.04, and **d** *P*_*r*_ = 11.32 and *T*_*r*_ = 1.16. Source data are provided as a Source Data file.

Because the network model aims to predict the fluid behavior at the level of molecular clusters, we compare its cluster size distribution to that of supercritical water in the top row of Fig. 4. In each regime, good agreement is observed between the network model and MD data. This agreement confirms our hypothesis that the network model can predict the clustering in supercritical water and, importantly, that the microscopic structure of supercritical fluids can be analyzed as a complex network of isotropic intermolecular interactions. Notably, because of the short lifetime of H-bonds at supercritical conditions^44^, their existence is not a prerequisite for a network analysis to be applied to these systems. Therefore, the links in these networks have an isotropic character and depend only on the intermolecular distance, but not on the orientation. Because these underlying physical networks are expected to be general, one can apply the proposed complex network model to reproduce quantities such as cluster size distributions and cluster fraction at different thermodynamic conditions in supercritical fluids. To further emphasize this, we extend our analysis by comparing predictions of the network model against experimental results by Bernabei et al.^10^ As shown in Fig. 5, cluster size distributions obtained using Hill’s energy criterion and predictions by the network model reproduce experimental data for a range of conditions that span the supercritical phase.

We proceed by analyzing the degree distribution, *n*_*d*_(*k*), which describes the connectivity of clusters, as shown in the bottom row of Fig. 4. The degree of a molecule, *k*, is defined by the number of its neighbors: two molecules neighbor each other if their interaction satisfies Hill’s energy criterion. It can be seen that for liquid-like conditions, where molecules are closely packed, the degree distribution is extended to larger values (${{{{{{{\mathcal{W}}}}}}}}=-\!0.13$, Fig. 4d). Because only molecules near the cluster boundary have a low degree of connectivity, in the high-density liquid-like state, a peak exists in *n*_*d*_(*k*) (labeled with an arrow at *k* = 2 in Fig. 4d) that moves to lower degree values with increasing temperature. In contrast, at gas-like conditions (${{{{{{{\mathcal{W}}}}}}}}=0.13$, Fig. 4f), molecules have higher kinetic energy and, thus, fewer interactions with other molecules in the system. Fewer interactions among molecules, in turn, lead to smaller clusters and a more compact degree distribution that peaks at *k* = 0. Similar to the cluster size distribution, the degree distribution is self-similar along isopleths of ${{{{{{{\mathcal{W}}}}}}}}$ in the extended supercritical region. For each ${{{{{{{\mathcal{W}}}}}}}}$, Fig. 4 depicts four thermodynamic state points (represented with different shades of blue), where the connectivity of the physical system remains invariant.

While the connectivity in the MD system evolves with the Widom self-similarity ${{{{{{{\mathcal{W}}}}}}}}$, the network model exhibits the same connectivity behavior when the link density *z* is varied. We compare these two systems for a range of conditions in the bottom row of Fig. 4. The connectivities predicted by the network model and the MD data are in good agreement for all three regimes. Based on these comparisons, we show that the network model is capable of predicting the density and distribution of molecular interactions of supercritical water. This observation suggests that the network model elucidates the mechanism of self-organization in supercritical fluids, not only at the system level, but also at the microstate level, where pair interactions between molecules define clusters.

**Fig. 6:**
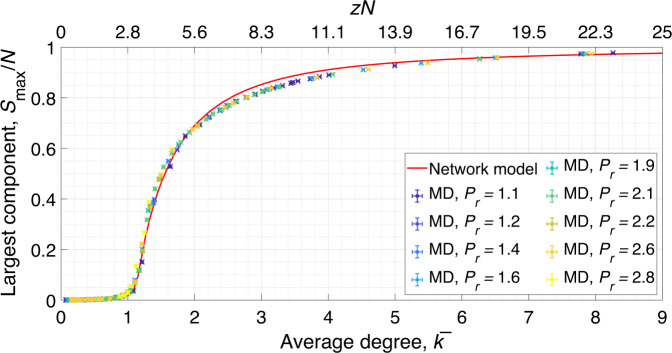
Relative size of the largest component as a function of average degree in the system. The network model is shown in red, while MD simulation data are represented with symbols colored based on the isobar. Error bars represent one standard deviation. The model captures the cluster evolution during the Widom line crossover. Source data are provided as a Source Data file.

Importantly, the network model may be employed to accurately predict the transition of supercritical fluids across the Widom line. In assessing this capability, we consider the evolution of the two systems as a function of the average degree, $\bar{k}$. This quantity can be interpreted as the average number of molecules in the first hydration shell. The size of the largest component in the system, ${S}_{\max }$, contains information about the percolation transition of the network, i.e., the structural crossover between liquid-like and gas-like states of supercritical fluids. When this cluster percolates the system, it also contains the majority of molecules present. The relative size of the largest cluster is defined as the fraction of molecules that belong to this cluster^56^. In Fig. 6, we show that the network model predicts the behavior that is observed from the MD solution when crossing the Widom line. Remarkably, the crossover for supercritical fluids does not change its behavior far from the critical point. This result confirms the generality of our network model in predicting the behavior of supercritical water.

### System dynamics

By extending the analysis of statistical properties, we proceed to examine the dynamics of the network model and to compare that dynamics to MD data. For this, we evaluate the model based on its predictions of molecular exchanges between clusters, which provides understanding of why the cluster size and degree distributions evolve across the Widom line.

**Fig. 7:**
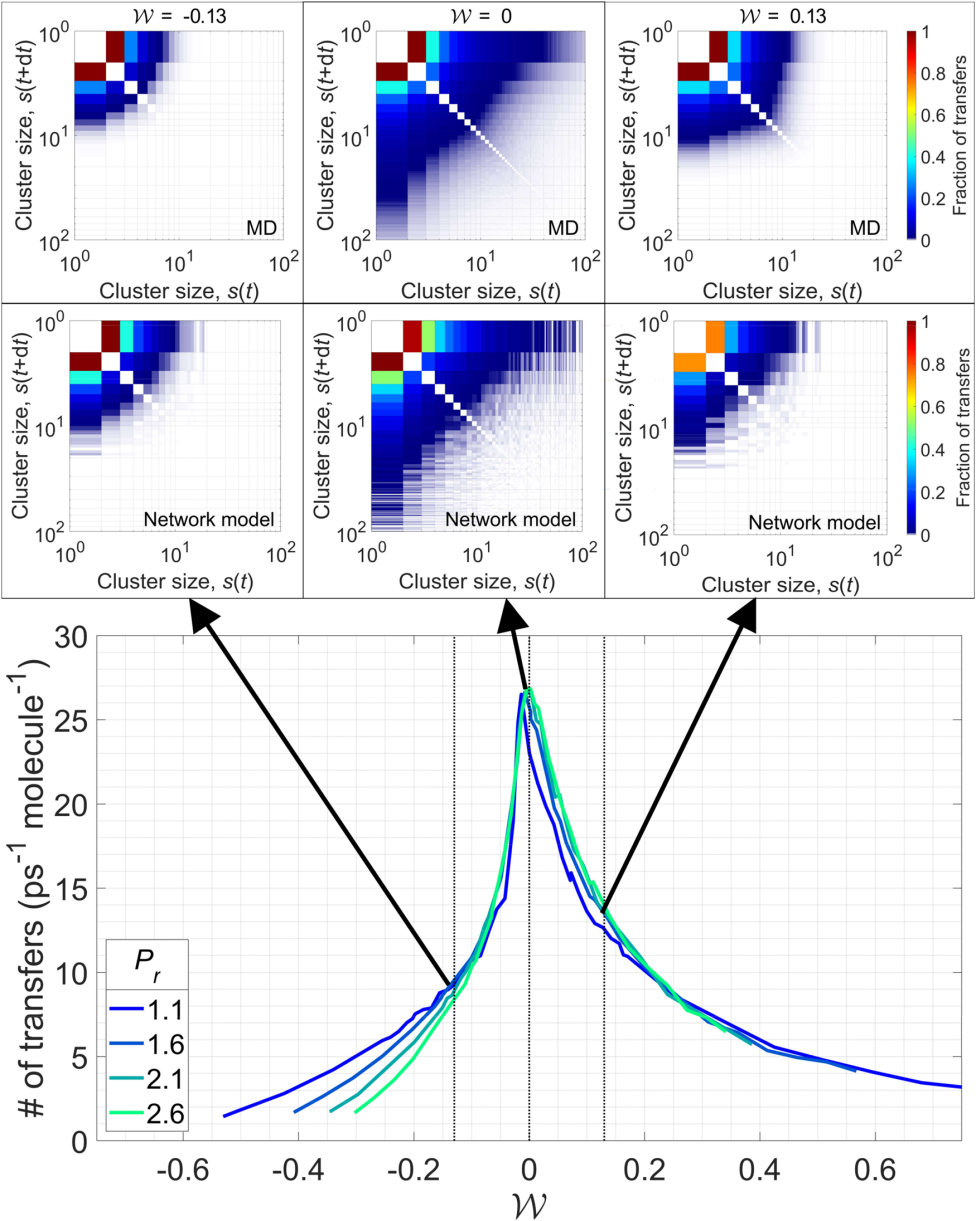
Molecular transfers between clusters. Molecular transfers rate peaks along the Widom line. **(inset)** Transfer matrices (with time increment *dt* = 2.5 ps) show that molecular exchanges are localized at a given scale: largest cluster in the liquid-like state (${{{{{{{\mathcal{W}}}}}}}}=-0.13$) and small clusters in the gas-like state (${{{{{{{\mathcal{W}}}}}}}}=0.13$). Along the Widom line (${{{{{{{\mathcal{W}}}}}}}}=0$), molecular exchanges occur among clusters of all scales. Transfer matrices for the network model replicate dynamics of fluid system. Source data are provided as a Source Data file.

Figure 7 shows that the molecular transfer rate peaks near the Widom line for all conditions considered. This peak obtains a consistent magnitude deep into the supercritical phase. Two balanced processes define the peak in molecular transfers: the entropically-favored fragmentation of clusters into gas-like molecules and the energetically-favored agglomeration of clusters into liquid-like assemblies^57^. Away from the Widom line, one of these processes dominates and the number of transfers significantly decreases. In the liquid-like state, the system’s energy is minimized by the existence of one large cluster that contains the majority of the fluid molecules (${{{{{{{\mathcal{W}}}}}}}}\,=\,-0.13$ in Fig. 4). The molecules that are enclosed within the core of the cluster are unlikely to transfer to another (smaller) cluster and, therefore, the number of molecular exchanges at liquid-like conditions is low. In contrast, the gas-like state is characterized by molecules with high kinetic energy and very few clusters, thereby maximizing the entropy of the system. Hence, the rarity of clusters and the high kinetic energy of fluid molecules make cluster exchanges improbable in the gas-like state. Evidence for the localization of these molecular transfers is found in the cluster transfer matrices (insets of Fig. 7), which quantify the number of molecular transfers between two subsequent time steps as a function of cluster size. Along the Widom line, where previously a scale-free cluster size distribution was found, we also find molecular exchanges among clusters of all sizes.

**Fig. 8:**
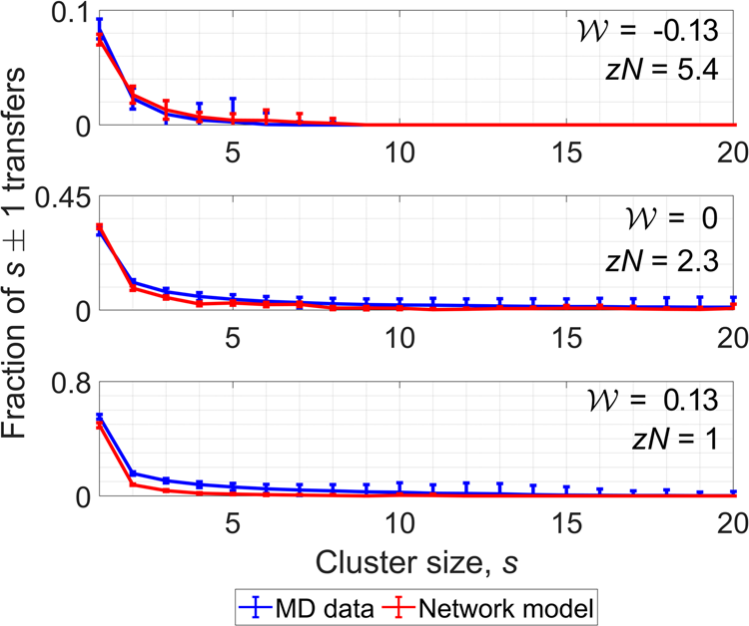
Single-molecule transfers between clusters. The distribution of single-molecule transfers peaks for the smallest clusters, especially at high temperatures where no large clusters exist in the supercritical phase. The network model can predict the fraction of single-molecule transfers in supercritical water. The error bars represent one standard deviation, computed over 2500 MD trajectory frames. Source data are provided as a Source Data file.

To evaluate the performance of the network model in predicting the cluster dynamics, in Fig. 7, we also compare the transfer matrices of the model with those obtained by analyzing molecular clusters in the supercritical phase. Because the network model has no notion of time, the network iterations are assumed to represent pseudo-time stepping in order to calculate the fraction of transfers in this system. Both the network model and the supercritical fluid system exhibit similar transfer distributions. Single-molecule transfers, which are defined as the number of clusters of size *s* that transition into size *s* ± 1 as a fraction of the total number of cluster transfers, dominate both systems. The majority of single-molecule transfers occurs due to agglomeration of unbound molecules into two-molecule clusters or due to fragmentation of two-molecule clusters, as shown in Fig. 8. There are fewer single-molecule transfers among clusters of increasing size. The model accurately predicts the distribution of these molecular exchanges as a function of cluster size. In agreement with the transfer matrices, the tail of the single-molecule transfer distributions extends to larger clusters only near the transition between liquid-like and gas-like behavior. The fraction of single-molecule transfers among the smallest, two-molecule clusters is the greatest in the gas-like regime, where no large clusters exist. Based on these dynamical measures, we conclude that supercritical fluids not only organize in complex network structures, but they also exhibit complex network dynamics.

## Discussion

We have identified self-similarity in the molecular structure for pure fluids and used this finding to show that supercritical fluids behave as complex networks. In a fluid beyond the critical pressure, molecules assemble into clusters of various sizes via a process that depends on local energetics. The main idea of this work is to formulate these clusters as components of a complex network of intermolecular interactions. In this physical network, each molecule represents a node that is connected to several other molecules, and the network’s links are defined based on Hill’s energy criterion. To mathematically describe the microscopic structure and dynamics of supercritical fluids, we showed that a complex network model can predict this behavior in terms of only few parameters. In contrast to previous lattice-based models of supercritical fluids, the presented complex network model does not prescribe orientation or density to the molecular interactions in the fluid. Therefore, it can capture both static and dynamic response of supercritical fluids as evidenced by direct comparison to MD simulations and experiments.

We were able to reach the above insight by extending Hill’s energy criterion to molecular species, which defined the cluster formation mechanism in supercritical fluids purely via physics-based energy arguments. Moreover, we proposed the Widom self-similarity, ${{{{{{{\mathcal{W}}}}}}}}$, to collapse the supercritical phase space. Finally, we demonstrated that the cluster size and degree distributions can be represented via a complex network model, whose link density *z* can be expressed in terms of experimental observables, *T* and *P*. This model also captures the molecular exchanges among clusters as observed from MD data.

The hidden-variable network model introduces the molecular fitness, which was sampled from a distribution. Physically, this fitness parameter can be related to the ability of a molecule to interact with neighboring molecules based on their relative energy balance. Therefore, the fitness landscape of the complex network model is analogous to the energy landscape that is traversed by each molecule to reach a local energy minimum^58^. Saykally and collaborators^59,60^ have experimentally characterized this energy landscape and the geometric structure for small clusters in liquid water. Further theoretical work is needed to characterize the molecular fitness in the context of supercritical fluids to generalize this network model.

Both the extension of Hill’s criterion to molecular species and the network model present new opportunities to connect the microscopic structure of the supercritical phase to thermodynamic and transport properties. The proposed network model can provide the cluster size distribution or the connectivity in the supercritical phase, thereby forming the foundation for developing constitutive models for supercritical fluids.

## Methods

Molecular dynamics (MD) simulations were performed to investigate clustering in supercritical water. The MD data was obtained with the Large-scale Atomic/Molecular Massively Parallel Simulator (LAMMPS) software package^61,62^ using a reactive force field (ReaxFF) potential for hydrogen and oxygen. The ability of this force field to predict the structure of supercritical water has previously been validated against simulations and experiments^40,41^. Further validation is provided in Supplementary Figs. 1–3, where the ReaxFF water model is shown to closely replicate both macroscopic (density and enthalpy) and microscopic (radial distribution functions) properties of water. The ability of ReaxFF water to replicate the cluster size distributions based on the experiments of Bernabei et al.^10^ (Supplementary Fig. 4) further justifies the use of this force field over other models. The isothermal-isobaric ensemble (NPT) was employed to study state points spanning reduced pressures between *P*_*r*_ = 1.1 and 2.8 and reduced temperatures from *T*_*r*_ = 0.46–1.9. For thermodynamic output data to converge, all simulations were performed with 8,544 water molecules in a cubic simulation box with periodic boundary conditions.

The equations of motion were integrated with the time-reversible measure-preserving Verlet integrator. A total simulation time of 250 ps was computed with a timestep of 0.25 fs. The presented results were averaged over 187.5 ps. Charge equilibration was done with the QEq method^63^ that used a Taper cut-off radius of 10 Å and an equilibration tolerance of 10^−6^*e*.

The network visualizations in Fig. 2 were created with the vis.js visualization library^64^.

### Reporting summary

Further information on research design is available in the Nature Portfolio Reporting Summary linked to this article.

## Supplementary information

Supplementary Information

Reporting Summary

## Data Availability

Source data are provided with this paper. The MD trajectories of supercritical water are permanently preserved in the Stanford Digital Repository at: 10.25740/nb780fd1620. The data that support the findings of this study are available from the corresponding author upon request. Source data are provided with this paper.

## Source data

Source Data

## Code availability

The MD simulations were performed with the open-source software LAMMPS. Code for the hidden-variable network model and scripts for the cluster analysis are available from the corresponding author upon request.
