# Supplementary material for: Supercritical fluids behave as complex networks
Source: Nat Commun. 2023 Apr 10;14:1996. doi: 10.1038/s41467-023-37645-z (PMC10083177; doi:10.1038/s41467-023-37645-z)
Supplement: Supplementary file 1 — Supplementary Information [file 41467_2023_37645_MOESM1_ESM.pdf]

Supplementary Information for “Supercritical fluids behave as complex networks”

Filip Simeski[1] **and Matthias Ihme**[1,2,*]

[1]Department of Mechanical Engineering, Stanford University, Stanford, CA 94305, USA
[2]Department of Photon Science, SLAC National Accelerator Laboratory, Menlo Park, CA 94025, USA
[*]mihme@stanford.edu

Supplementary Note 1: Cluster definition

Hill[1] defined microscopic clusters of monoatomic gases based on atomic energies. He proposed that two monoatomic molecules, i and j, form a bound pair if the negative of their interaction potential energy is greater than their relative kinetic energy:

$$E_{\text{pot},ij} + E_{\text{kin},ij} < 0. \tag{1}$$

This clustering approach is purely physics-based and requires no input parameters other than those already used by the MD model. Yet, it has not been previously defined for molecular species that are computationally represented by multiple atomic sites[2]. Here, we extend the mathematical formulation of Hill's energy criterion to polyatomic molecules. To this end, we consider two polyatomic molecules: i and j. These molecules form a bound pair if their interaction satisfies Hill's energy criterion (Equation 1) and each set of molecules that are interconnected through bound pairs forms a cluster. When calculating the interaction potential energy of molecular pairs, only the non-bonded interactions of ReaxFF are considered. These interactions represent the van der Waals and Coulombic forces. The non-bonded van der Waals interactions are given by the Morse potential with a Taper correction[3].

In our implementation, the atomic interaction energies as proposed by the ReaxFF formulation should be recast as molecular interaction energies. Because there are three atoms in each water molecule, there are nine different atomic pair interaction potential energies that contribute to the total interaction potential energy of a molecular pair. Adding these atomic interactions reduces the number of interaction potential energies from $\frac{1}{2}N_{\text{atom}}^2$ to $\frac{1}{2}(N_{\text{atom}}/3)^2$, where N_{atom} is the total number of atoms in the system:

$$E_{\text{pot},ij} = \sum_{\zeta\in i}\sum_{\theta\in j}\left(E_{\text{vdW},\zeta\theta} + E_{\text{Coul},\zeta\theta}\right) . \tag{2}$$

The relative kinetic energy of two molecules in the simulation, i and j, is computed with respect to the molecular center of mass (COM) velocity. Each water molecule can be studied as a three-body system, whose conservation of momentum gives:

$$\mathbf{u}_\zeta = \frac{\sum_{\zeta\in i}^3 m_\zeta \mathbf{v}_\zeta}{\sum_{\zeta\in i}^3 m_\zeta}. \tag{3}$$

Then, the relative kinetic energy of the molecular COMs is

$$E_{\text{kin},ij} = \frac{1}{2} m_{\text{H}_2\text{O}} \|\mathbf{u}_i - \mathbf{u}_j\|^2 . \tag{4}$$

The final step of extending Hill's energy criterion to molecular systems is comparing the interaction potential energies of molecules against their relative kinetic energies, according to Equation 1. If this inequality is true, we assign both molecules to the same cluster, otherwise they are not considered bound. Mathematically, the clustering algorithm at each time instance, t, has the following steps:

1. *Cluster initialization*: Assign a random molecule i to cluster $C_m^{(t)}$ with $m = 1, 2, ..., N_{\text{cluster}}^{(t)}$.
2. *Energy calculation*: For molecule i and all other molecules (j), compute the interaction potential energy, $E_{\text{pot},ij}$, and the relative kinetic energy, $E_{\text{kin},ij}$, according to Equations 2 and 4, respectively.
3. *Cluster assignment*: If these two energy values satisfy Hill's criterion (Equation 1), assign molecule j to the cluster, $C_m^{(t)}$, where molecule i belongs:

$$C_m^{(t)} := \left\{ j \mid (\exists\, i \in C_m^{(t)}) \left[E_{\text{pot},ij} + E_{\text{kin},ij} < 0\right] \right\}. \tag{5}$$

Repeat steps 2-3 for molecule j.

4. *Restart*: When there is no other molecule j that belongs to $C_m^{(t)}$ according to Equation 5, consider the next cluster $C_{m+1}^{(t)}$ and start over from step 1 with a random molecule that has not been assigned yet.

While the details of this derivation concern the Morse potential and Coulombic interactions, the approach is general enough to be used with other interatomic potentials. All pair interactions between molecules are included in the potential energy, $E_{\mathrm{pot},ij}$, and therefore, this quantity obtains a very large negative value when two molecules are connected by a H-bond[4]. Therefore, the network formed by any reasonable H-bond definition is inherently also formed by Hill's energy criterion. Yet, unlike H-bond networks, which can only be defined for H-bonded fluids, a network based on Hill's energy criterion can be established for any fluid comprised of interacting molecules.

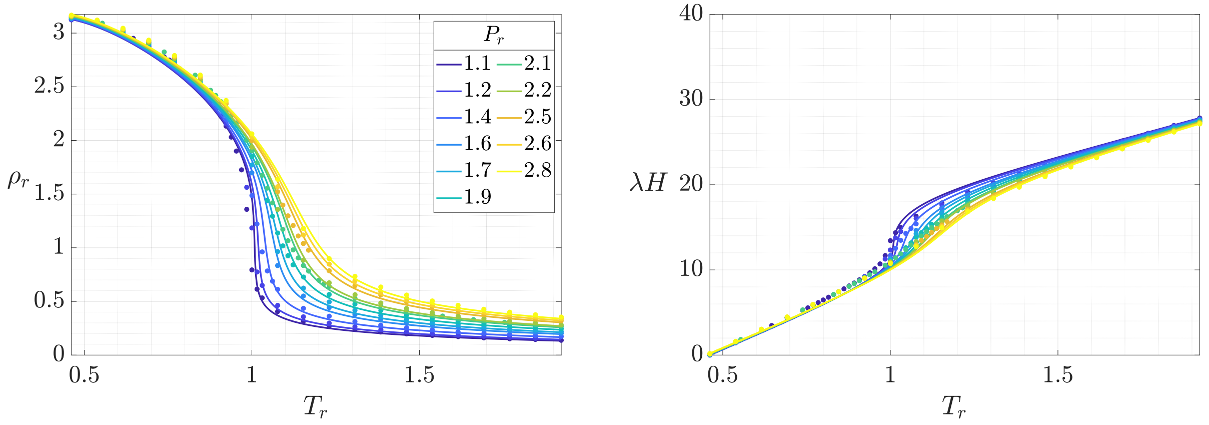

(a) Density

(b) Enthalpy

Supplementary Figure 1. Comparisons of **(a)** density and **(b)** enthalpy of the ReaxFF water model (symbols) and experimental data from the NIST Webbook[5] (solid lines). The agreement extends across the whole range of temperatures and pressures that are investigated in our work.

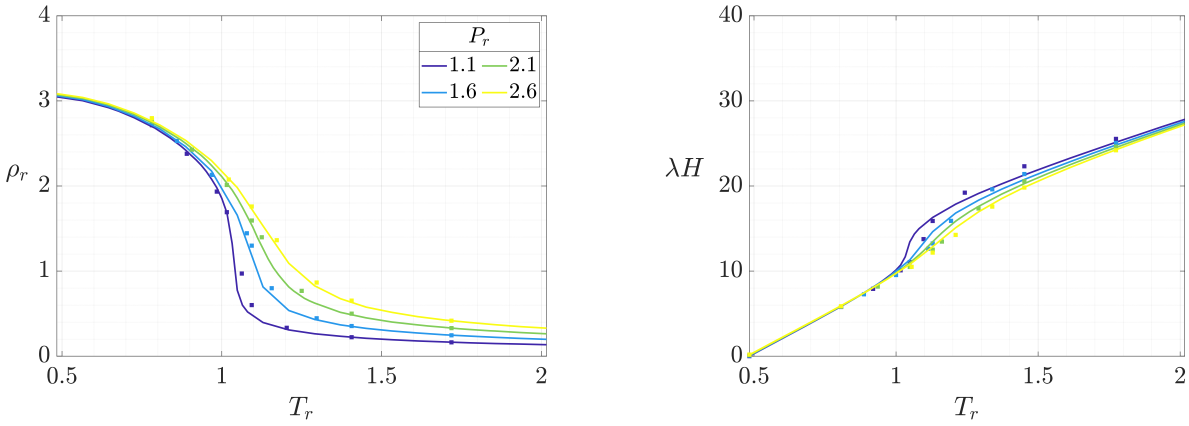

(a) Density **(b)** Enthalpy

Supplementary Figure 2. Comparisons of **(a)** density and **(b)** enthalpy of the ReaxFF water model (solid lines) and MD data based on TIP4P/2005 water (symbols) across the whole range of temperatures and pressures that are investigated here. TIP4P/2005 is a standard nondissociative and rigid water model.

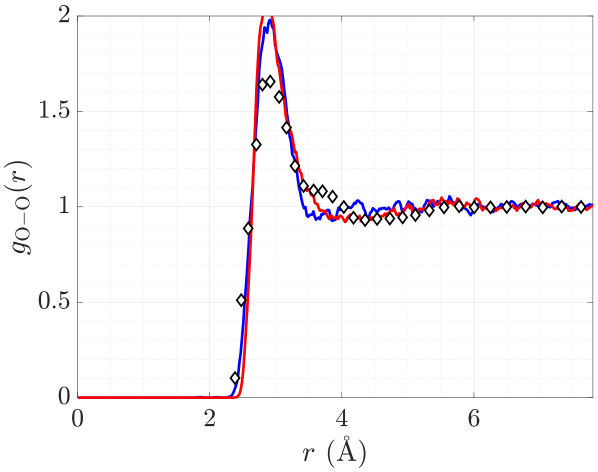

(a) O-O radial distribution function

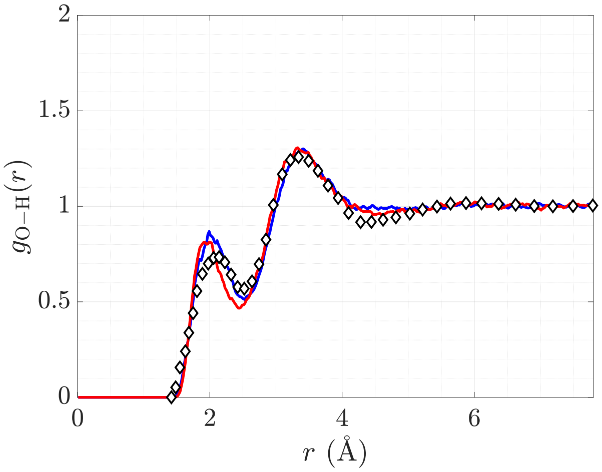

(b) O-H radial distribution function

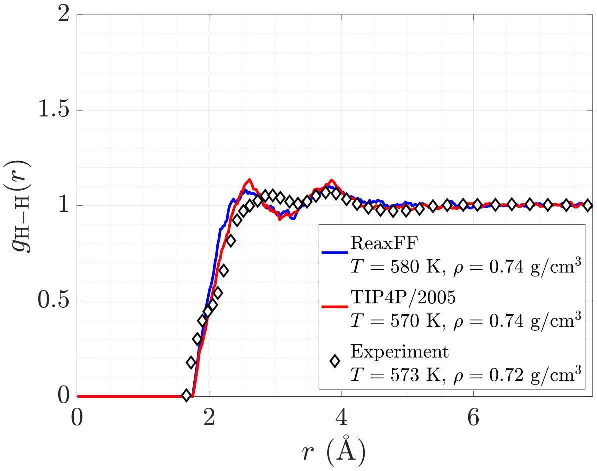

(c) H-H radial distribution function

Supplementary Figure 3. Radial distribution functions (RDFs) for supercritical water. Comparison of the NDIS-97 dataset[6], ReaxFF simulations of bulk water, and TIP4P/2005 simulations of bulk water in terms of the **(a)** O-O RDF, **(b)** O-H RDF, and **(c)** H-H RDF.

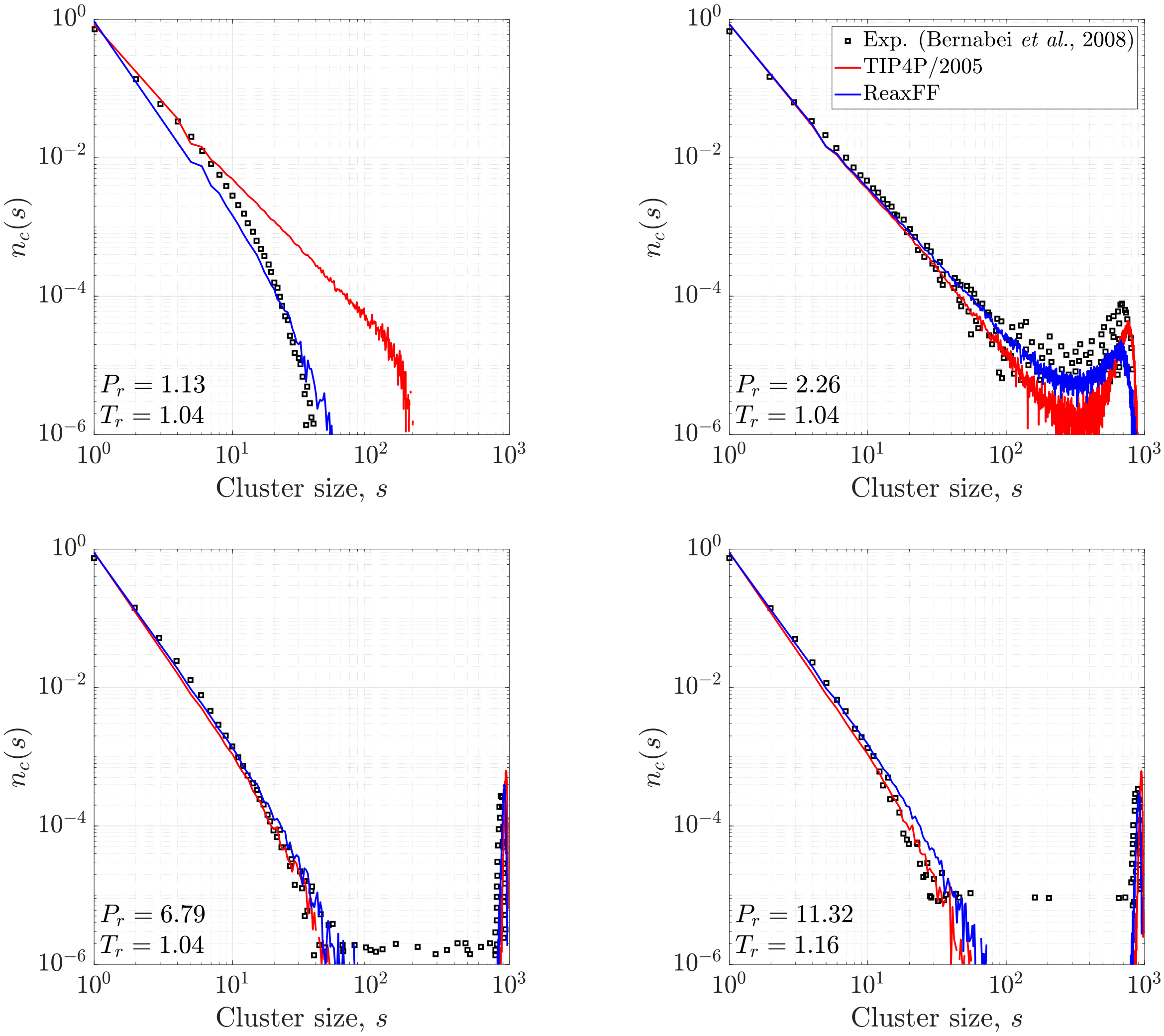

Supplementary Figure 4. Comparison of ReaxFF and TIP4P/2005 water models with experiments in terms of the normalized cluster size distributions. The experimental data is from Bernabei *et al.*[7] The deviation of the cluster size distribution obtained with TIP4P/2005 in the first panel is due to the shift in the critical point of this force field.

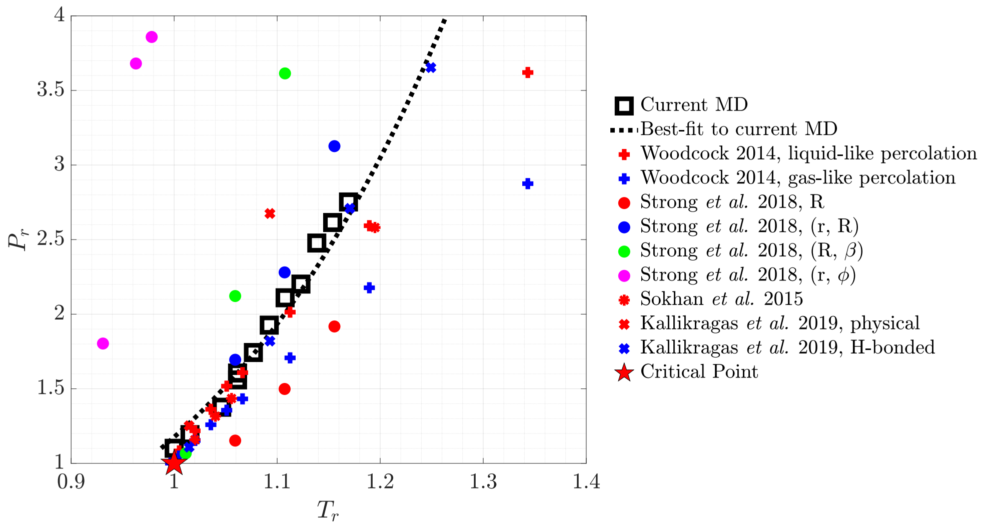

Supplementary Figure 5. Loci of percolation line are identified from the cluster size distributions, similarly to the approach taken by Kallikragas and Svishchev[8]. Our percolation line loci agree well with those of other computational and experimental studies[8–11].

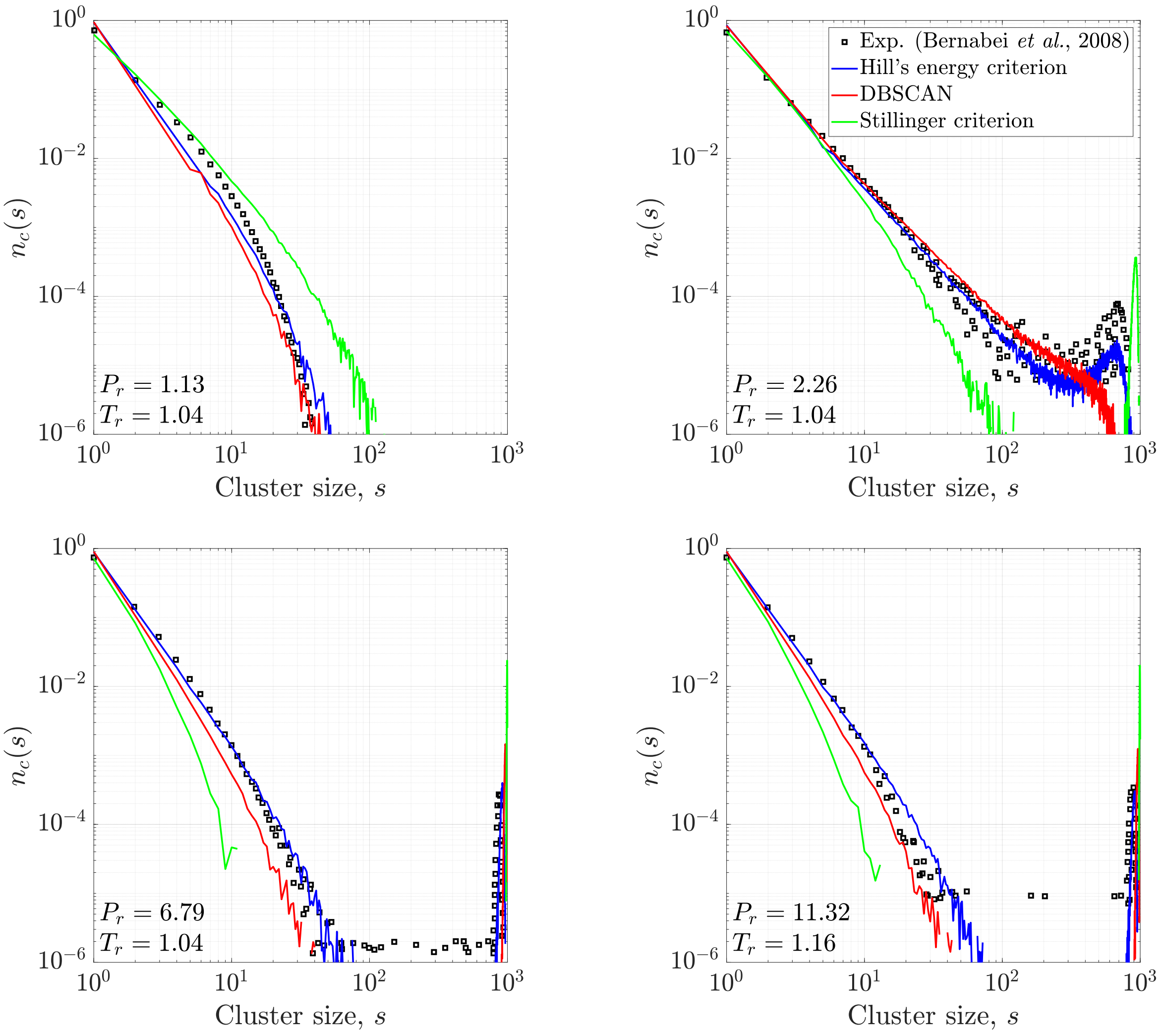

Supplementary Figure 6. Comparison of cluster size distributions that are produced by the clustering algorithm based on Hill's energy criterion, which is used in this work, and two other commonly used clustering algorithms: DBSCAN[12] and the Stillinger criterion[13]. The experimental data is from Bernabei *et al.*[7] The Stillinger criterion fails to accurately capture the cluster sizes. While the other two clustering approaches produce similar cluster size distributions, Hill's energy criterion does not depend on any tuning parameters, while DBSCAN has two parameters (here, $\varepsilon = 3.4$ Å and minPts $= 4$ from the geometry of H-bonds).

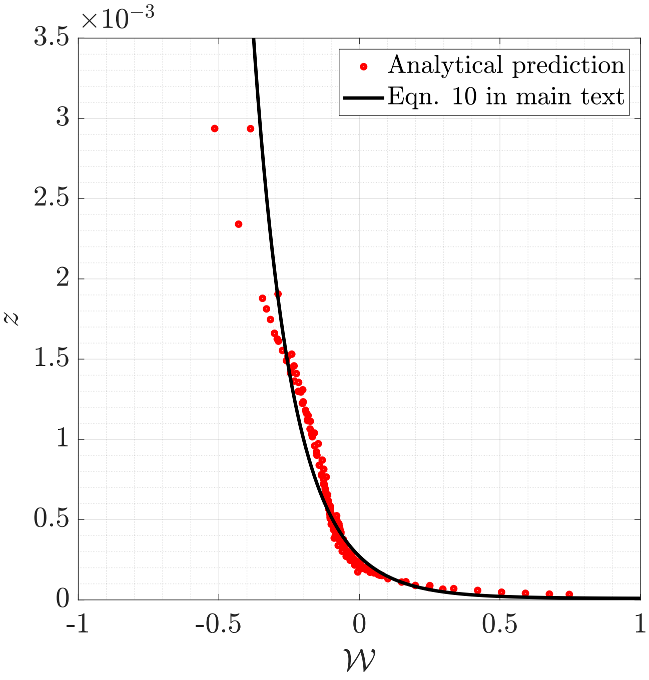

Supplementary Figure 7. Curve fit for the link density z as a function of the Widom self-similarity, $\mathcal{W}$. The analytical predictions (red symbols) for z are directly calculated from MD data via Equations 7 and 8 in the main text, where the total number of links, M, is assumed a known quantity for the physical system. The black line represents a regression (Equation 10 in the main text), which connects z directly to thermodynamic conditions.

Supplementary References

1. Hill, T. L. Molecular clusters in imperfect gases. *J. Chem. Phys.* **23**, 617 (1955).
2. Škvor, J. & Nezbeda, I. Percolation line and response functions in simple supercritical fluids. *Mol. Phys.* **109**, 133–139 (2011).
3. Chenoweth, K., van Duin, A. C. T. & Goddard, W. A. ReaxFF reactive force field for molecular dynamics simulations of hydrocarbon oxidation. *J. Phys. Chem. A* **112**, 1040–1053 (2008).
4. Swope, W. C., Andersen, H. C., Berens, P. H. & Wilson, K. R. A computer simulation method for the calculation of equilibrium constants for the formation of physical clusters of molecules: Application to small water clusters. *J. Chem. Phys.* **76**, 637–649 (1982).
5. Lemmon, E. W., McLinden, M. O. & Friend, D. G. Thermophysical properties of fluid systems. In Linstrom, P. J. & Mallard, W. G. (eds.) *NIST Chemistry WebBook, NIST Standard Reference Database Number 69* (National Institute of Standards and Technology, Gaithersburg MD, 20899, 2022).
6. Chialvo, A. A., Cummings, P. T., Simonson, J. M., Mesmer, R. E. & Cochran, H. D. Interplay between molecular simulation and neutron scattering in developing new insights into the structure of water. *Ind. Eng. Chem. Res.* **37**, 3021–3025 (1998).
7. Bernabei, M., Botti, A., Bruni, F., Ricci, M. A. & Soper, A. K. Percolation and three-dimensional structure of supercritical water. *Phys. Rev. E* **78**, 021505 (2008).
8. Kallikragas, D. T. & Svishchev, I. M. Percolation transitions of physically and hydrogen bonded clusters in supercritical water. *J. Mol. Liq.* **290**, 111213 (2019).
9. Woodcock, L. V. Gibbs density surface of water and steam: 2nd debate on the absence of van der Waals' "critical point?. *Nat. Sci.* **6**, 411–432 (2014).
10. Strong, S. E., Shi, L. & Skinner, J. L. Percolation in supercritical water: Do the Widom and percolation lines coincide? *J. Chem. Phys.* **149**, 084504 (2018).
11. Sokhan, V. P., Jones, A., Cipcigan, F. S., Crain, J. & Martyna, G. J. Molecular-scale remnants of the liquid-gas transition in supercritical polar fluids. *Phys. Rev. Lett.* **115**, 117801 (2015).
12. Sander, J., Ester, M., Kriegel, H.-P. & Xu, X. Density-based clustering in spatial databases: The algorithm GDBSCAN and its applications. *Data Min. Knowl. Discov.* **2**, 169–194 (1998).
13. Stillinger Jr., F. H. Rigorous basis of the Frenkel-Band theory of association equilibrium. *J. Chem. Phys.* **38**, 1486 (1963).
