# Supplementary material for: Supercritical fluids behave as complex networks
Source: Nat Commun. 2023 Apr 10;14:1996. doi: 10.1038/s41467-023-37645-z (PMC10083177; doi:10.1038/s41467-023-37645-z)
Supplement: Supplementary file 2 — Reporting Summary [file 41467_2023_37645_MOESM2_ESM.pdf]

nature portfolio

Corresponding author(s): Matthias Ihme
Last updated by author(s): Mar 17, 2023

Reporting Summary

Nature Portfolio wishes to improve the reproducibility of the work that we publish. This form provides structure for consistency and transparency in reporting. For further information on Nature Portfolio policies, see our Editorial Policies and the Editorial Policy Checklist.

Statistics

For all statistical analyses, confirm that the following items are present in the figure legend, table legend, main text, or Methods section.

n/a	Confirmed	
x	☐	The exact sample size (n) for each experimental group/condition, given as a discrete number and unit of measurement
x	☐	A statement on whether measurements were taken from distinct samples or whether the same sample was measured repeatedly
x	☐	The statistical test(s) used AND whether they are one- or two-sided<br>*Only common tests should be described solely by name; describe more complex techniques in the Methods section.*
x	☐	A description of all covariates tested
x	☐	A description of any assumptions or corrections, such as tests of normality and adjustment for multiple comparisons
x	☐	A full description of the statistical parameters including central tendency (e.g. means) or other basic estimates (e.g. regression coefficient) AND variation (e.g. standard deviation) or associated estimates of uncertainty (e.g. confidence intervals)
x	☐	For null hypothesis testing, the test statistic (e.g. F, t, r) with confidence intervals, effect sizes, degrees of freedom and P value noted<br>*Give P values as exact values whenever suitable.*
x	☐	For Bayesian analysis, information on the choice of priors and Markov chain Monte Carlo settings
x	☐	For hierarchical and complex designs, identification of the appropriate level for tests and full reporting of outcomes
x	☐	Estimates of effect sizes (e.g. Cohen's d, Pearson's r), indicating how they were calculated

Our web collection on statistics for biologists contains articles on many of the points above.

Software and code

Policy information about availability of computer code

Data collection	Molecular dynamics simulations were performed with the open-source software package LAMMPS using a reactive force field potential for hydrogen and oxygen.
Data analysis	Analysis of the results using Hill's method was done using scripts that were developed in-house with algorithms presented in the manuscript. The visualizations of the complex networks were created with the vis.js visualization library.

For manuscripts utilizing custom algorithms or software that are central to the research but not yet described in published literature, software must be made available to editors and reviewers. We strongly encourage code deposition in a community repository (e.g. GitHub). See the Nature Portfolio guidelines for submitting code & software for further information.

Data

Policy information about availability of data

All manuscripts must include a data availability statement. This statement should provide the following information, where applicable:
- Accession codes, unique identifiers, or web links for publicly available datasets
- A description of any restrictions on data availability
- For clinical datasets or third party data, please ensure that the statement adheres to our policy

Source data are provided with this paper. The MD trajectories of supercritical water are permanently preserved in the Stanford Digital Repository at: https://

doi.org/10.25740/nb780fd1620. The data that support the findings of this study are available from the corresponding author upon reasonable request.

Human research participants

Policy information about studies involving human research participants and Sex and Gender in Research.

Reporting on sex and gender	We are reporting results from a computational study, not involving any human research participants
Population characteristics	We are reporting results from a computational study, not involving any human research participants
Recruitment	We are reporting results from a computational study, not involving any human research participants
Ethics oversight	We are reporting results from a computational study, not involving any human research participants

Note that full information on the approval of the study protocol must also be provided in the manuscript.

Field-specific reporting

Please select the one below that is the best fit for your research. If you are not sure, read the appropriate sections before making your selection.

[x] Life sciences [ ] Behavioural & social sciences [ ] Ecological, evolutionary & environmental sciences

For a reference copy of the document with all sections, see nature.com/documents/nr-reporting-summary-flat.pdf

Life sciences study design

All studies must disclose on these points even when the disclosure is negative.

Sample size	For each operating condition (p/T) we performed one MD simulation that was time-advanced over 250ps for statistically stationary conditions. Each MD-simulation used 8544 water molecules; the operating conditions spanned reduced pressures between Pr = 1.1 and 2.8 and reduced temperatures from Tr = 0.46 to 1.9.<br>Simulations using the hidden-variable network model were performed using the same operating conditions and system size to facilitate direct comparisons with MD data.
Data exclusions	No data was excluded from the analysis.
Replication	We verified our MD simulations by comparing against results using different force-fields and experimental data for validation. Results from these verification studies are provided as supplementary information.
Randomization	No randomization of the data was used; all computational data, obtained from the MD simulations was used for the analysis
Blinding	No blinding of the data was used; all computational data, obtained from the MD simulations was used for the analysis

Reporting for specific materials, systems and methods

We require information from authors about some types of materials, experimental systems and methods used in many studies. Here, indicate whether each material, system or method listed is relevant to your study. If you are not sure if a list item applies to your research, read the appropriate section before selecting a response.

Materials & experimental systems

n/a	Involved in the study	
[x]	[ ]	Antibodies
[x]	[ ]	Eukaryotic cell lines
[x]	[ ]	Palaeontology and archaeology
[x]	[ ]	Animals and other organisms
[x]	[ ]	Clinical data
[x]	[ ]	Dual use research of concern

Methods

n/a	Involved in the study	
[x]	[ ]	ChIP-seq
[x]	[ ]	Flow cytometry
[x]	[ ]	MRI-based neuroimaging
